# Empowering older adults: evaluating the impact of a smartphone education app on independent living

**DOI:** 10.3389/fpubh.2024.1403978

**Published:** 2024-08-16

**Authors:** Ye-Shin Woo, Ga-in Shin, Hae Yean Park

**Affiliations:** ^1^Occupational Therapy Department, College of Software Digital Healthcare Convergence, Yonsei University, Wonju, Republic of Korea; ^2^Department of Rehabilitation Medicine, Seoul National University Hospital, Seoul, Republic of Korea; ^3^National Traffic Injury Rehabilitation Research Institute, National Traffic Injury Rehabilitation Hospital, Yangpyeong, Republic of Korea

**Keywords:** older adults, smartphone, education, usage, eye-tracker

## Abstract

**Purpose:**

As science and technology advance, older people’s ability to use smart devices in their daily living is becoming more demanding. This study addresses the increasing use of smartphones by the older adults, who often struggle with technology due to lack of competence. We developed an educational app tailored for older adults users and compare its effectiveness with existing educational videos.

**Methods:**

An app was created based on the learning characteristics of the older adults, using the ADDIE model, and compared with traditional video education. It involved six participants aged 65 or older, and convenience sampling method was used, evaluating the app and video through usability assessments and eye tracking. Quantitative and qualitative analyzes were conducted with focus groups under the researcher’s control.

**Results:**

The app received higher usability scores than the video in content, motivation, and interaction. Eye tracking showed users paid more consistent attention to the app.

**Conclusion:**

The smartphone app facilitates learning for the older adults without the constraints of time and place, improving their quality of life and technology skills. Eye tracking can be instrumental in future app development for this demographic.

## Introduction

The proportion of people aged 65 and older in Korea increased from 7.2% in 2000 to 15.7% in 2020, entering an aged society. In 2050, Korea is expected to become the country with the highest proportion of older adults among OECD (Organization for Economic Cooperation and Development) countries (1). As the average life expectancy increases due to the development of medical technology and the improvement of standards of living, interest in spending the old age in a healthy and meaningful way is increasing (2). Activities of daily living (ADL) are important indicators for predicting the healthy life and quality of life of older adults (3).

The recent development of information and communication technology is bringing about various changes in ADL (4). The advent of mobile living services necessary for daily life, such as public transportation reservation/reservation, Internet banking, and online shopping, makes it possible to purchase goods without physical effort or time and space limitations and to conveniently use services necessary for daily life such as banks and public transportation (5). Many experts predict that as the number of smartphone users increases, more diverse apps and mobile services will emerge (6). Living services are now being demanded more (7).

A survey on the use of living services using smart devices for older adults showed that the rate of using life information services (70.4%) was the highest, followed by financial transaction services (37.4%), e-commerce services (33.8%), and public services (15.3%), and the rate of using financial transactions and e-commerce services in which financial transactions are carried out online was low (8). The use of mobile living services necessary for daily life can make older adults’ daily life more convenient, and the proportion of older adults using smartphones is steadily increasing (9). Thus, the use of mobile living services can be an important means to maintain an independent life for older adults.

Although the possibility of supporting a safe and independent life has increased as most older adults have smartphones (10), older adults who are not familiar with and do not understand how to use smartphones may have difficulty acquiring and using various information (11). Currently, smartphone education programs for older adults offered by local governments are limited in the duration of education and the number of participants due to financial constraints. Thus, the level of learning is not enough for older adults to use smartphones in their daily lives (12). Therefore, smartphone education videos have been distributed online to overcome these limitations. However, older adults who are not accustomed to online learning face difficulties learning using videos (13). In particular, as aging progresses, much cognitive effort and a large amount of learning are required to learn smartphone functions due to deterioration in physical and cognitive functions, such as intelligence and memory, which are required to learn new functions, and deterioration in vision and visual resolution (14, 15).

Considering these points, education based on apps is one of the effective education methods for older adults. The advantages of using apps are as follows: First, it is possible to provide customized education tailored to individual needs. Second, education can be done without being restricted by time and space. Third, learning motivation and sense of efficacy can be improved through success experiences such as appropriate feedback and rewards. Fourth, most apps are operated using a touch screen, which lowers cognitive barriers through visual, auditory, and tactile stimulation, thereby supporting smartphone learning more effectively (16). Lastly, mobile apps can make educational interventions more convenient and applicable because they can be freely downloaded and used at any time.

Therefore, this study aimed to develop a smartphone education app suitable for the characteristics of older adults and to develop the education area step by step to make older adults’ daily life more convenient. The “use of public transportation” was selected as the priority function to be developed. The use of public transportation is one of the important instrumental activities of daily living (IADL). Previous studies have shown that “movement to the community” is the most important area for self-management for older adults with chronic diseases, following “physical activity” and “ADL and IADL management,” and “knowing how to use public transportation” is the most suitable and necessary skill for older adults with chronic diseases who want to participate in activities in the community (17). The 2020 survey on the condition of older adults showed that public transportation accounted for 71.2% of the means of transportation that older adults usually use when going out (18). Older adults prefer to use public transportation when moving, and it can be confirmed that this leads to active use of public transportation (19). This study introduces the “using public transportation” educational app developed in Korea and presents the effectiveness of the educational program app through objective evaluation and subjective usability evaluation using an eye-tracking device.

## Materials and methods

### Procedure

**Figure 1:**
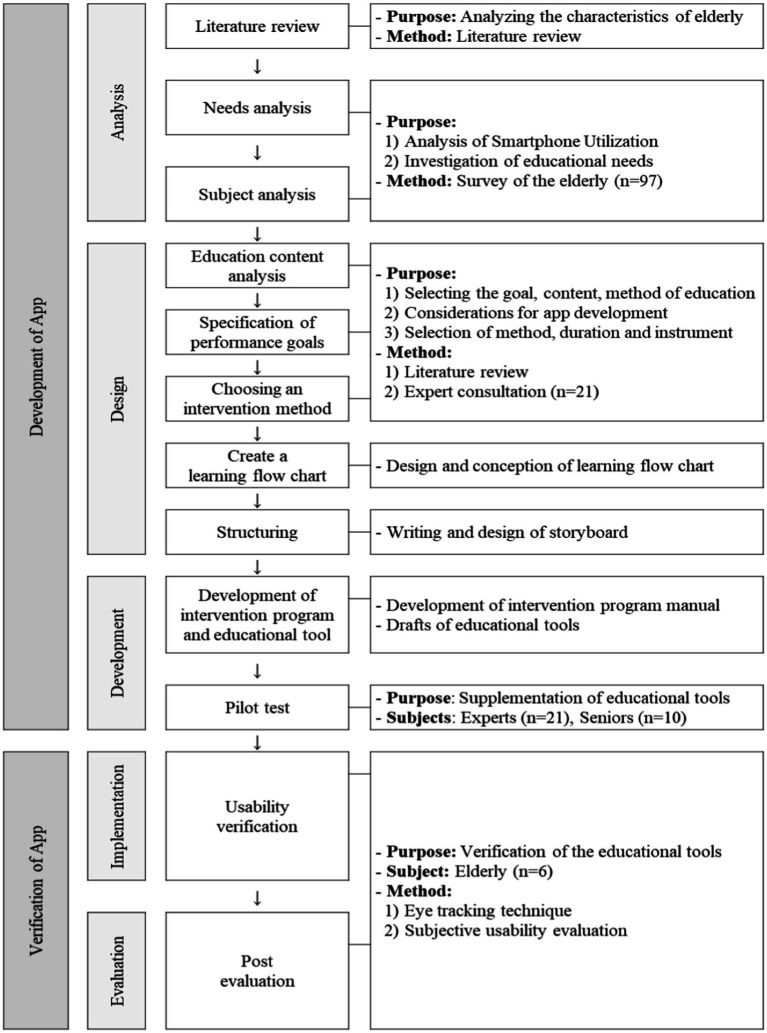
ADDIE model.

This study developed a program based on the ADDIE (Analysis, Design, Development, Implementation, and Evaluation) model (20), which is commonly used in educational program development. The research contents were largely divided into “development of smartphone education application” and “verification of education application” (Figure 1).

#### Analysis

A smartphone education demand survey was conducted targeting older adults to determine the justification of the study.

#### Design and development

Through a literature review, considerations for the production of an educational app for older adults were identified, and a learning flowchart and storyboard for the educational app were designed to develop the app.

#### Implementation

A 1:1 pilot test was conducted targeting experts and older adults to identify and supplement modifications and improvements. The intervention method, intervention period, and evaluation tool of the smartphone education program were produced as an educational manual based on the results of the expert consultation.

#### Evaluation

The usability of the smartphone education app was quantitatively analyzed using an eye-tracking device to verify the effectiveness of the smartphone education program. The significance of the smartphone education program was demonstrated by evaluating the task performance related to smartphone use after education.

### Participants

People aged 55 years or older who use a smartphone, have no experience in using the app used for training in this study, and voluntarily expressed their intention to participate in the experiment were included in this study (Table 1). The convenience sampling method was used. Quantitative and qualitative analyzes were conducted with focus groups under the researcher’s control. Since gaze movement is affected by brain imbalance (21), this study was limited to right-handed subjects. Eye tracking was restricted when wearing thick glasses. All subjects participated in this study after providing informed consent.

**Table 1:** General information of participants.

PID	Sex	Year of birth	Education	Spouse or not	Inmate or not	Duration of usage
P01	F	1959	Elementary school	Y	Spouse	5 years ↑
P02	F	1958	Middle school	Y	Spouse and son	10 years ↑
P03	F	1957	Elementary school	Y	Spouse	5 years ↑
P04	F	1955	Elementary school	Y	Spouse	5 years ↑
P05	F	1941	University	N	–	10 years ↑
P06	F	1954	Elementary school	Y	Spouse	5 years ↑

### Smartphone education application

#### Flow chart

**Figure 2:**
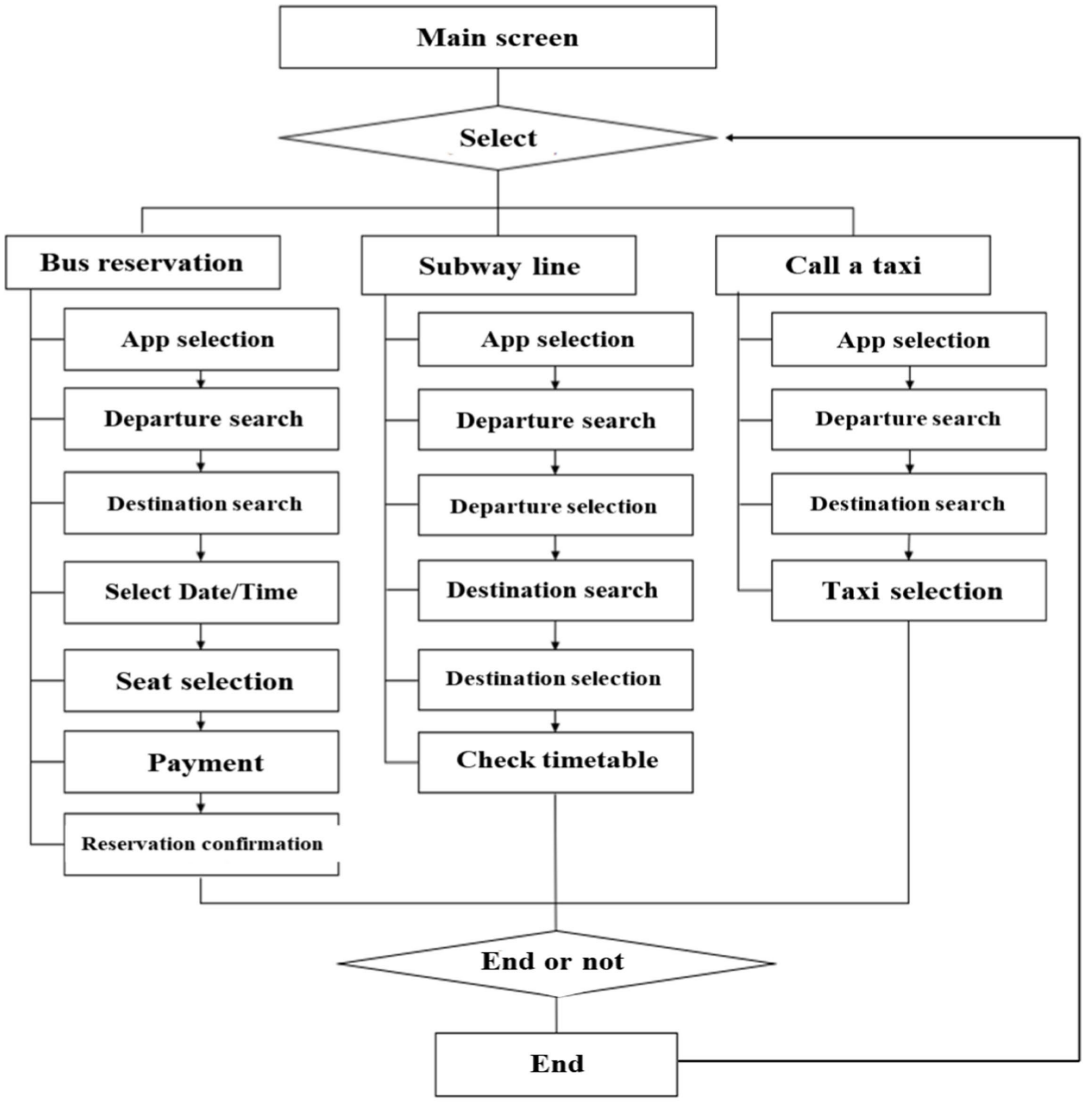
Flow chart of smartphone education.

The smartphone education app has two screens: a main screen and an item selection screen. On the main screen, the learning area (e.g., “using public transportation”) is presented, and the learners select an item from the item selection screen (e.g., “Bus reservation,” “Subway line,” and “Call a Taxi”) by touching the screen. After selecting an item to learn, images, descriptions, voices, and feedback are provided on how to use it. Thus, each stage can be learned by oneself. The flowchart of the educational content is shown in Figure 2. Each stage is composed of the steps required to use the app on a smartphone. For example, participants must practice seven steps to use a bus reservation app. These are “app selection,” “departure location search,” “destination search,” “date/time selection,” “seat selection,” “payment,” and “reservation confirmation.”

#### Storyboard

**Figure 3:**
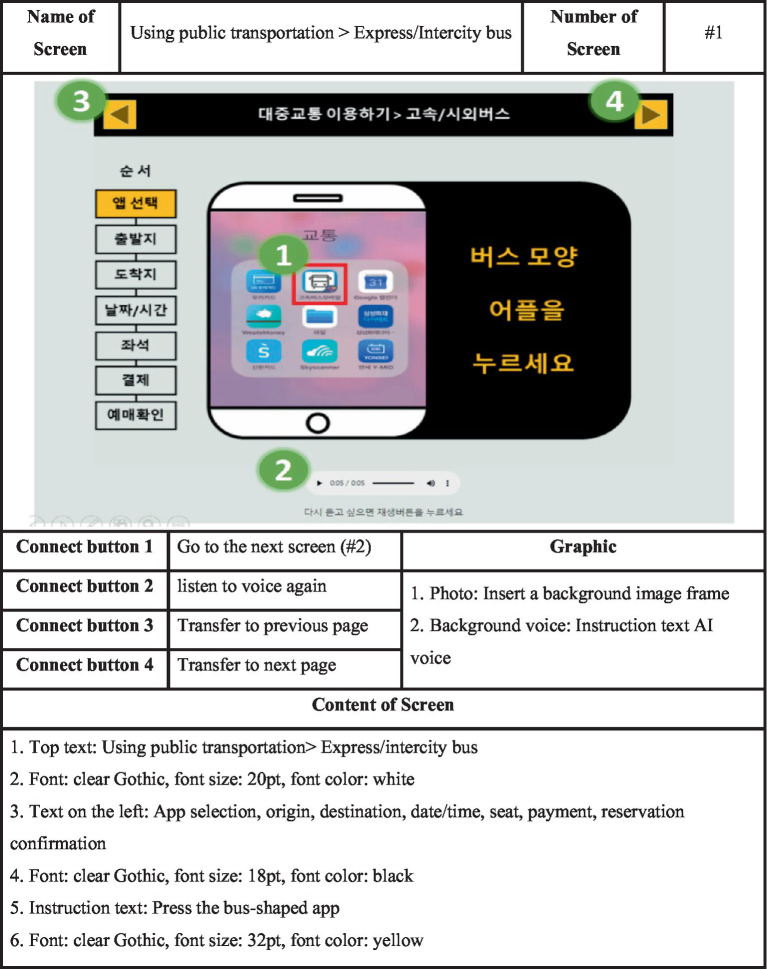
Storyboard.

The storyboard is the blueprint required to create a smartphone education app. Much attention should be paid to design and composition, such as images, size and shape of letters, and voice, to help older learners use educational tools efficiently. A storyboard helps present the content to be implemented (interface style, color, etc.) in a consistent and unified way and facilitates communication between developers and the development process. In this study, the design for the production of the educational tool was created based on the contents derived from the analysis and design stages (Figure 3).

### Assessments and measures

#### Subjective usability rating scale

An evaluation scale based on the mobile app evaluation sheet used by Jang (22) and Kim (23) was reconstructed and used to evaluate existing smartphone education videos and the smartphone education app. The evaluation scale consisted of 18 items in five areas, and each item was assessed on a 5-point Likert scale (Table 2).

**Table 2:** Subjective usability rating scale.

Item	Content of question	Number of questions
Content	Content accuracy, level, effectiveness, and achievement of learning goals	4
Motivation	Attention, satisfaction, and confidence	3
Monitor	Placement, color, consistency, amount of content, text, and images	6
Interaction	Learning degree identification, feedback, and learning speed control	3
Defect	Accuracy and error	2
Total		18

#### Eye tracker

In this study, eye The eye movements of all participants were recorded using an eye-tracking device (Tobii TX300, Stockholm, Sweden). Eye-tracking devices determine which elements the user pays attention to and how interested they are (24, 25) through eye movements input through specific visual information. Eye-tracking devices infer the result of the user’s cognitive thinking based on physiological response data such as gaze fixation and saccades. Fixation is maintaining the gaze on a specific object (images, sentences, etc.). The area where the gaze is fixated is the area of interest (AOI), and the fixation duration can be used to measure the concentration. The frequency of gazing was analyzed. Usually, the fixation duration is 100 ms or 0.1 s (26), and the standard differs depending on the type of information presented. The eye-tracking technique provides various visual output methods for measurement results. A heatmap was used, which is a visual representation of the user’s gaze fixation during eye tracking using a color gradient. The color gradient ranges from green to red; the more the fixation, the more red the color. This helps compare the overall tendency of information (images, sentences, etc.) preferred by subjects.

### Experimental protocol

#### Test procedure

**Figure 4:**
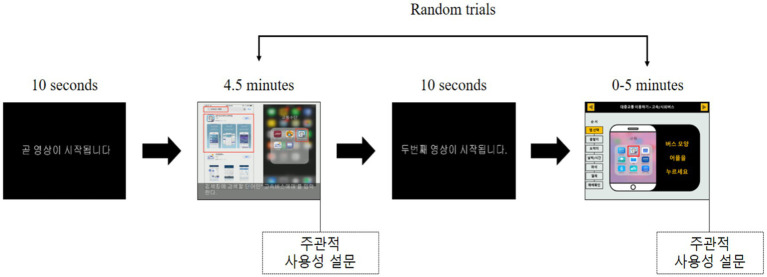
Test procedure.

Before the experiment, the researcher checked the experimenters’ health condition and explained the process of measurement using the eye-tracking device. Before measurement, elements that might be eye-tracking obstacles were removed from the subject as much as possible. Before use, the eye-tracking device was calibrated for the nine areas presented on the screen. This experiment consisted of six steps: “Instruction”-“Smartphone training video”-“Survey”-“Instruction”-“Smartphone training tool”-“Survey,” and the required time was about 20–30 min. The first directive was presented for 10 s, and the content of the directive was “Video will start soon.” The first task was to watch educational videos on smartphones. After watching the video, a survey was conducted. After the survey, the second directive was presented for 5 s, and the content of the directive was “The second video will start soon.” The second task is to use the smartphone education tool. After that, a survey was conducted. The task order was presented randomly for each experimenter to reduce the effect on the playback order (Figure 4).

#### Data analysis

The average difference in the subjective usability evaluation between the existing smartphone training videos and the developed smartphone training tool was assessed using a paired-sample t-test. Statistical analysis was performed using SPSS Statistics 25 (IBM, Armonk, NY, United States). A heatmap and gaze movement path (gaze) based on data on fixation and instantaneous eye movements (saccades) of the gaze were used. A plot was used to analyze the attentional tendency of each educational medium, measure the number of times the user’s gaze stays in the designated AOI and the gaze time, and infer the objective results of the cognitive thinking of the experimenters.

## Results

### Results of subjective survey

**Figure 5:**
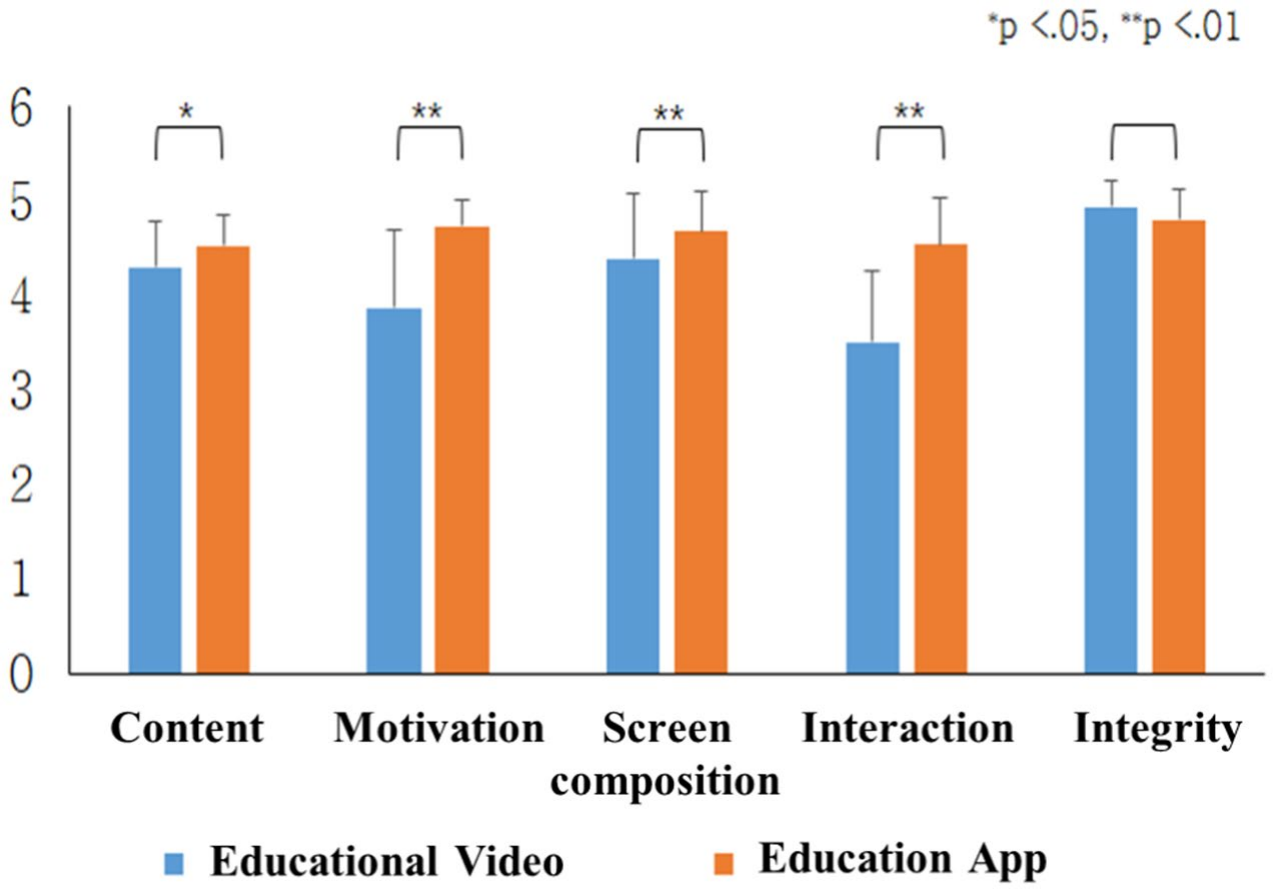
Results of subjective survey.

For subjective usability evaluation, a questionnaire was conducted on five areas (content, motivation, screen composition, interaction, and integrity). The comparison between the subjective usability of the existing smartphone training videos and the developed smartphone training app showed that the subjective usability scores of the smartphone training app were significantly higher than those of the existing smartphone training videos in content (*p* = 0.014), motivation (*p* = 0.005), screen composition (*p* = 0.032), and interaction (*p* = 0.003), but not in integrity (*p* = 0.083) (Figure 5). The total score was 20.96 points for the smartphone education videos and 23.23 points for the smartphone education app, which indicated a significantly higher subjective usability evaluation score of the developed education app (*p* = 0.002).

### Result of quantitative eye tracking

#### Setting of AOI

**Figure 6:**
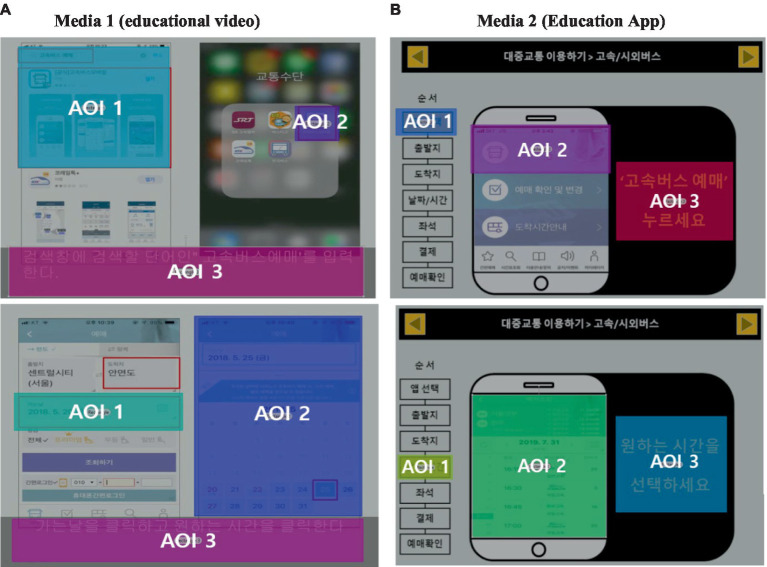
Result of setting AOI by media.

To regions of interest (AOI) to be included in the analysis were set for each medium. In this study, as the area where education was in progress is the analysis range, text and picture areas corresponding to the education stage for each medium were set as AOI. Figure 6 illustrates three areas of interest. The AOI 1 represents the instructional text area, AOI 2 is the image content area, and AOI 3 encompasses interactive elements. The AOI area designated by the authors refers to the area that subjects need to focus on.

#### Comparison of fixation ratio by media

**Figure 7:**
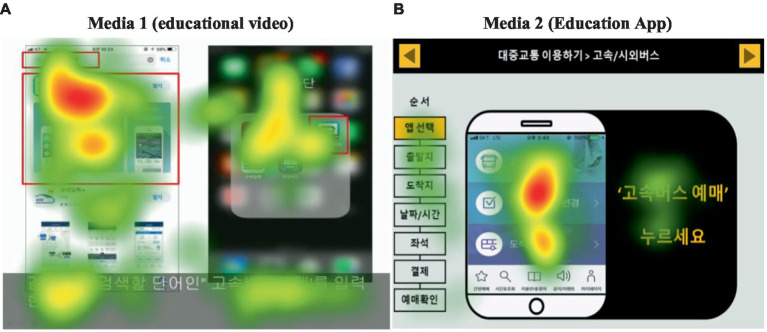
Result of fixation ratio by media: heat map.

The result of analyzing the heat map for the fixation tendency for each medium is shown in Figure 7. In Media 1 (educational video), the eyes were distributed over the entire area, and it could be seen that the eyes were also distributed in sections outside of the educational content. However, in Media 2 (education app), it could be seen that the gaze is relatively distributed in the center.

Table 3 shows fixation counts for each medium. The total number of fixations for more than 100 ms (0.1 s) for each medium was analyzed. The analysis of the total number of gaze fixations (total fixation count), the number of accurate gaze fixations on the target area (accuracy fixation count), and the accurate fixation rate (accuracy rate) in each stage according to the medium showed that, except for stage 1 (selection of apps) and stage 6 (payment), the accuracy fixation rates of Media 2 were higher than those of Media 1. In the case of Media 1, the accuracy rates were inconsistent as the steps progressed, and the accuracy rates of Media 2 were maintained at 80% as the steps progressed, showing consistent results.

**Table 3:** Result of fixation ratio by media.

	Media 1 (educational video)		%	Media 2 (education app)		%
	Total (count)	Accuracy (count)		Total (count)	Accuracy (count)	
Stage 1	469	304	64.8	625	178	28.5
Stage 2	1,184	509	43.0	352	309	87.8
Stage 3	550	165	30.0	351	315	89.7
Stage 4	978	743	76.0	753	646	85.8
Stage 5	1,061	778	73.3	325	262	80.6
Stage 6	1,658	1,629	98.3	1,802	1,490	82.7
Stage 7	754	478	63.4	721	644	89.3
Total	6,654	4,606	69.2	4,929	3,844	78.0

#### Comparison of visit duration by media

**Figure 8:**
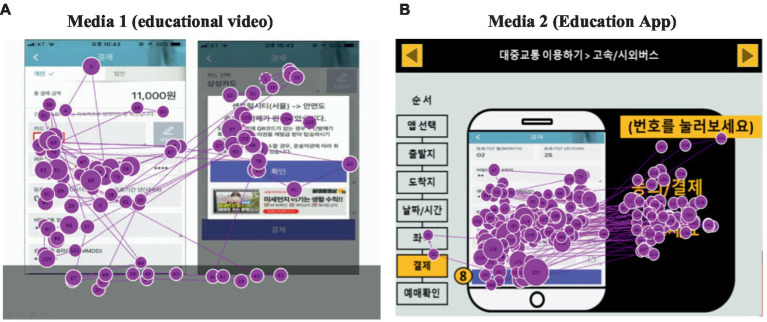
Result of gaze movement by media: gaze plot.

Figure 8 shows the gaze plot for gaze movements by media. The place where the gaze moves quickly is indicated by a line, and the size of the circles changes according to the visit duration. The sizes of the circles in Media 2 are larger than those in Media 1. Furthermore, more gaze movements can be seen in Media 2.

Table 4 shows the visit duration for each medium. The time to accurately gaze at the area related to the educational content was 1552.4 s in Media 1, accounting for 57.1% of the total learning time of 2508.6 s. In Media 2, the time was 1599.2 s, accounting for 71.7% of the total required time of 2242.1 s. In the case of learning using Media 2, the rate of gaze time was at least 1.1 times and at most 3.1 times higher than that of Media 1. Media 2 took more than 70% of the total learning time in all areas except for step 1 in the area related to education. Media 1 took more than 80% in the area related to education in step 6. The examination time was less than 70% in all stages except for the above.

**Table 4:** Result of visit duration by media.

	Media 1 (educational video)		%	Media 2 (education app)		%
	Total (second)	Accuracy (second)		Total (second)	Accuracy (second)	
Stage 1	157.9	94.4	59.8	289.0	71.8	24.8
Stage 2	430.3	158.2	36.2	166.6	139.9	83.9
Stage 3	202.4	56.1	27.7	162.5	139.2	85.6
Stage 4	360.3	249.4	69.2	342.8	270.2	78.8
Stage 5	404.6	255.22	63.1	137.2	102.6	74.8
Stage 6	657.8	570.0	86.7	808.4	613.2	75.9
Stage 7	295.3	169.1	57.3	335.6	262.3	78.2
Total	2508.6	1552.4	57.1	2242.1	1599.2	71.7

### Qualitative analysis of effectiveness in education program

After the completion of the mobile app-based smartphone education program, a focus group interview was conducted with six participants in the experiment to derive the following meaningful themes.

#### Areas of improvement after educational program

Most respondents reported gaining confidence and interest in learning after smartphone education. Additionally, they responded that it helped them conveniently use public transportation in their daily lives without having to contact their children to ask for help or wait in line and that the time to talk with their children or spouse about what they had learned increased.

① Enhancing the convenience of using public transportation.

“*I had to call my daughter every time to find out the train ticket time, but now it is so convenient to be able to check the train ticket time myself. It seemed to bother her daughter, but since she can do it by herself, she loves it too.*” (Subject 4)

“*It’s nice to be able to go on a trip without asking my children, and I think the most convenient thing is not having to wait in a long line.*” (Subject 5)

“*I could not go far because it was difficult to get around, but it’s nice to know in advance the time of the subway I want to take. Even if I cannot get on, it’s nice to be able to check what time the next car is.*” (Subject 6)

② Improvement of interest and confidence in learning.

“*I was scared when I said I was learning a smartphone, but after learning and practicing steadily, I gained confidence that I could do it. I wanted to ask if I could do it on my smartphone because I was speaking English while taking care of my grandchildren.*” (Subject 1)

“*After using it, it seemed to have a lot of fun and novel features, so I looked around for this and that. I want to learn other skills as well*” (Participant 2).

“*I did not do it perfectly, but after practicing several times, it seems to work to some extent.*” (Subject 3)

“*Before, I did not think of learning anything because I did not know anything, but now I have the confidence that I can do it too.*” (Subject 4)

③ Improving communication with spouse.

“*I boasted to her husband that now I could buy a train ticket by myself. My husband asks her daughter to buy a train ticket every time, but when I said I would, she liked it and asked me to let her know too.*” (Subject 6)

#### Improvement and future direction

The results of the practice through the smartphone education app were satisfactory, and all subjects responded that the practice content was helpful in actually using the public transportation app. Regarding the direction of future education, a common response was that they did not know what functions would be good to learn because they did not yet know what functions were available. Additionally, regarding functions involving financial transactions, they reported that they needed an intermediate inspection because they were afraid of doing it alone. They hoped that such smartphone education opportunities would be expanded.

① Provides additional information on useful features for the older adults.

“*I do not know what other functions of a smartphone exist besides public transportation. It would be nice if you could recommend a good function to learn.*” (Subject 1)

“*The functions I used on my smartphone were just making calls, KakaoTalk, and watching videos. It’s good because it’s convenient because I learned about public transportation, but I do not know because I have not used the other functions well.*” (Subject 6)

② Necessity of mid-progress inspection.

“*It worked well when I practiced, but it did not go well when I tried to do it alone. However, there was no one by my side to teach me, so I could not solve the problem.*” (Subject 3)

“*When I practice alone, I worry that money will go out because of my mistake. So I did not even do the card payment part and practiced until then.*” (Subject 6)

③ Expansion of accessibility to smartphone education programs.

“*There are times when you need to use it right away, but your smartphone is dead. At such times, I would go to a nearby young friend or a mobile phone store and ask for a solution. I wish there were more places around me where I could ask questions at any time.*” (Subject 2)

“*Because I work during the daytime, there were few opportunities to receive such education. I hope there will be more opportunities to receive education.*” (Subject 3)

## Discussion

In this study, a smartphone education program for the area of “using public transportation” was developed, considering the characteristics of older adults. The effectiveness of the education program was verified. “Using public transportation” is a representative ADL item for community mobility. Ensuring the mobility of older adults has an important meaning. Using smartphone apps related to public transportation plays an important role in promoting IADL performance, which is community mobility. Using smartphones is expected to play a positive role in the life of older adults by providing practical convenience for them to perform daily life and increasing their satisfaction accordingly.

As older adults’ smartphone ownership rate and the use of smartphones in daily life increase, older adults’ interest in smartphones is increasing. Using smartphones requires processing and learning, which puts a burden on older adults (14, 25, 27–30). Thus, it is necessary to enhance older adults’ interest in smartphone education, such as finding educational methods to reduce the burden of using smartphones for older adults to increase their smartphone usage ability in daily life.

In this study, a smartphone education app for older adults was developed, and its usability was verified, considering the barrier factors and solutions related to smartphone learning for older adults. The study results showed that, in the case of educational videos, the accuracy of gaze fixation and gaze time were high at the beginning of education but decreased as time passed, suggesting that maintaining attention for learning with educational videos is limited. Videos are hard to match the learning pace of individual users and contain a large amount of visual information. Therefore, in the case of older adults who have difficulty processing various visual stimuli at the same time, attention is thought to be lower in the later stages than in the early stages (31, 32). In contrast, in the case of the smartphone education app, the accuracy of gaze fixation and gaze time were low at the beginning of education but increased as time passed. In the case of older adults who were unfamiliar with education using the app, tension increased at the beginning of the education, but since behavioral responses to visual cues and voice explanations were required from the beginning to the end of the education, it was found that gaze fixation and gaze time were high and consistently maintained as time passed, and this is foreseeable. These results showed that the education method using the app could provide effective education to older adults.

In this study, the developed smartphone training app showed qualitatively and quantitatively higher usability and consistent attention than the existing smartphone training video. Furthermore, the visual clues and voice explanations included in the smartphone education app influenced the maintenance of attention by recognizing the learning content and inducing the touch action to move on to the next step. Previous studies have shown that when complex information needs to be presented to older adults, auditory and visual information are provided at the same time, and the learning effect is enhanced when the process of searching for information occurs to a minimum (33–37). This study is meaningful in that it quantitatively evaluated smartphone education app usability. The study findings help provide comprehensive information to prepare a smartphone education program for older adults.

This study has some limitations. First, in this study, only some areas of education were implemented because this study aimed to verify the usability of the smartphone education app and the effectiveness of the program. Thus, in future research, it is necessary to specify the smartphone education area by examining the smartphone functions necessary for the improvement of the daily life of older adults in addition to education on “using public transportation.” Second, this study verified the usability and effectiveness of the program for older adults without cognitive deficits over the age of 60. Thus, further studies are needed to increase the number of subjects and verify the effectiveness of the program according to age and educational level to increase the generalizability of the verification results. Additionally, it is necessary to find a way in which all older adults can collect and use appropriate information using smartphones by distinguishing between older adults who can receive an education using apps and those who need practical help. In summary, the study’s small sample size and short duration may limit the generalizability of the findings. Further research with a larger cohort and extended follow-up is needed.

## Conclusion

This study is significant in that it developed a mobile app-based smartphone education program that considers the characteristics of older adults to increase smartphone usage ability and objectified the usability of the educational app by using quantitative analysis methods along with subjective response evaluation. Smartphone education using mobile apps has significantly higher usability than educational videos. Furthermore, attention is great and maintained consistently. Mobile app-based smartphone education programs contribute to increasing the ability of older adults to use apps on their own. Moreover, they help induce older adults’ ADL and independent and active participation in society. Future initiatives should focus on expanding access to smartphone education programs for older adults, integrating intermediate checks to support continuous learning, and exploring additional functionalities that can further aid in independent living.

## Data availability statement

The original contributions presented in the study are included in the article/Supplementary material, further inquiries can be directed to the corresponding author.

## Ethics statement

The studies involving humans were approved by the Institutional Review Board (IRB) of Yonsei University College (IRB No. 1041849-202008-SB-100-2). The studies were conducted in accordance with the local legislation and institutional requirements. The participants provided their written informed consent to participate in this study.

## Author contributions

Y-SW: Conceptualization, Formal analysis, Investigation, Methodology, Project administration, Software, Visualization, Writing – original draft, Writing – review & editing. G-iS: Investigation, Writing – review & editing. HP: Funding acquisition, Supervision, Writing – review & editing.
