# Supplementary figures and images for: Empowering older adults: evaluating the impact of a smartphone education app on independent living
Source: Front Public Health. 2024 Aug 16;12:1403978. doi: 10.3389/fpubh.2024.1403978 (PMC11363426; doi:10.3389/fpubh.2024.1403978)

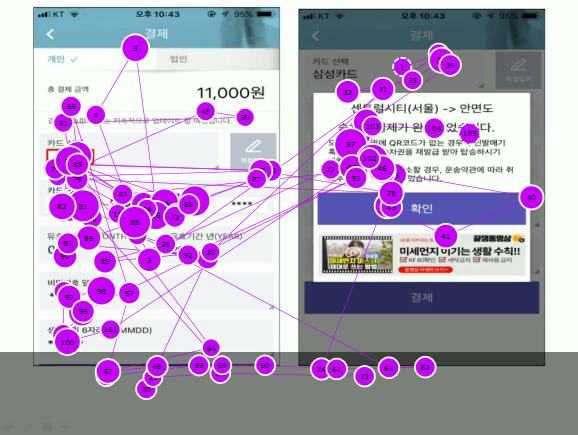

Supplement: Supplementary file 1 [file Image_1.TIF]

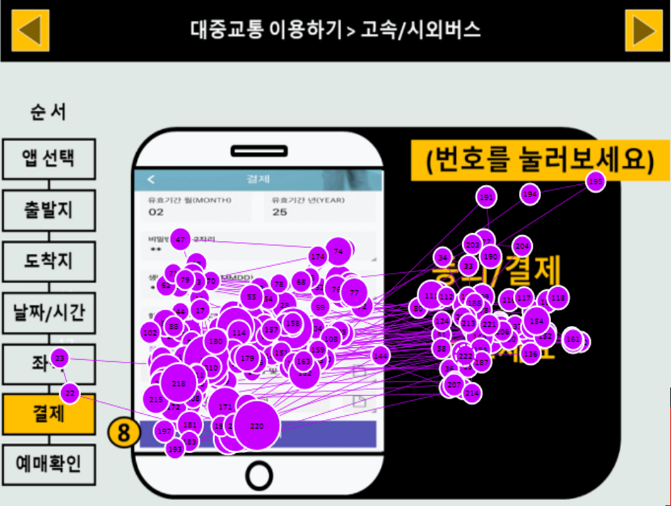

Supplement: Supplementary file 2 [file Image_2.TIF]
